# Observation of risk perception, knowledge and behaviour related to covid-19 in heart failure patients enrolled on a telecoaching program

**DOI:** 10.1186/s12911-026-03613-y

**Published:** 2026-06-22

**Authors:** Stefanie Rosner, Sarah M. Leiter, Teresa Trenkwalder, Amadea Erben, Patrick Fuchs, Christian Kloss, Wibke Reinhard, Katharina Knoll

**Affiliations:** 1 Department of Cardiovascular Diseases, School of Medicine and Health, Technical University of Munich, German Heart Centre Munich, TUM University Hospital, Munich, Germany; 2 Present Address: Health Care Systems GmbH (HCSG), Pullach i. Isartal, Germany; 3 DZHK (German Centre for Cardiovascular Research), Partner Site Munich Heart Alliance, Munich, Germany

**Keywords:** COVID-19, Chronic heart failure, Telemedicine, Telecoaching, Telenursing

## Abstract

**Background:**

Telemedical interventions have been shown to reduce mortality and morbidity and improve the quality of life of chronic heart failure (CHF) patients. During the Covid-19 pandemic remote coaching gained further importance; however, it is not clear if Covid-19-specific telecoaching has a long-lasting impact on behaviour change.

**Methods:**

Patients were pre-existing participants in a combined tele-monitoring and telecoaching programme for CHF. A total of 419 patients were assessed with a standardised questionnaire immediately before and three weeks after a COVID-19-specific telecoaching in April 2020, as well as eight months later. The aim of the study was to observe changes in knowledge and behaviour regarding COVID-19 risk reduction measures, number of medical contacts and self-perceived health risk over time following the telecoaching module.

**Results:**

After telecoaching, patients spontaneously recalled significantly more COVID-19-specific risk reduction measures rising from an average of 2.1 items prior to coaching to 2.5 at short- and long-term follow-up (*p* = 0.0002). The number of self-reported medical contacts were significantly lower at short-term than at long-term follow-up (30% vs. 42%, *p* = 0.0060 family doctor, 5% vs. 12%, *p* = 0.0014 hospital). CHF patients perceived themselves as low risk for a severe COVID-19 infection, and this perception did not change after telecoaching. No difference in social isolation or concern over time were noted.

**Conclusions:**

Our longitudinal observational study suggests a possible effect of a single COVID-19-specific telecoaching module on knowledge about the disease and complying with risk reduction measures, which seems to persist over time. These results should be interpreted with caution in the context of increasing public awareness and public health campaigns.

**Supplementary Information:**

The online version contains supplementary material available at 10.1186/s12911-026-03613-y.

## Introduction

Corona Virus Disease 2019 (COVID-19), the disease caused by the Severe Acute Respiratory Syndrome coronavirus 2 (SARS-CoV-2) [1], emerged in December 2019 in Wuhan, China, and rapidly spread globally, infecting about 700 million people worldwide [2] and causing up to 6.9 million deaths by Summer 2023. Although the majority of COVID-19 infections are asymptomatic or present mild, flu-like symptoms [3, 4], a significant proportion of patients develops severe respiratory illness, requiring hospitalisation and even ventilatory support [3, 4]. Elderly patients, men, and those with coexisting medical conditions, such as cardiovascular disease, hypertension and diabetes, are at higher risk for a severe clinical course [3, 5].

Prior to the universal rollout of vaccinations, a preventive strategy to reduce the spread of infection was implemented [6–8]. This strategy included isolation of suspected and confirmed cases, systematic monitoring and isolation of contacts, and finally, broad public health measures with physical distancing regulations, school closures, and travel restrictions [4, 6].

This social distancing tactic proves a challenge with chronically ill patients who require regular clinical follow-up. The risk of delaying treatments or missing early deterioration needs to be balanced with the risk of increased personal contacts and thus more opportunities of viral transmission [7]. During the peaks of the pandemic, government restrictions limited the access to specialist care [9]. Additionally, patients delayed or avoided seeking medical help during the early pandemic period due to fear of infection [10].

Heart failure is a chronic condition associated with frequent hospitalisations and high morbidity and mortality rates [11], requiring regular monitoring and continuous patient support. Disruption of care during the COVID-19 pandemic delayed implementation of prognostic relevant heart failure medication [12, 13]. In the attempt to ensure continuity of care and to prepare health care systems for the second wave of COVID-19, telehealth systems have been advocated [14, 15]. Telehealth programmes allow delivery of health care at physical distance, thus reducing community and nosocomial spread [15]. Besides providing continuity of care through virtual physician consultations, telehealth can motivate patients to improve health promoting behaviour through telecoaching [16].

In heart failure patients, telemedical interventions have been shown to reduce mortality and morbidity as well as improve quality of life [17–19]. In a Cochrane review, both non-invasive telemonitoring and telecoaching reduced all-cause mortality and heart failure related hospitalisations in heart failure patients [18]. Telecoaching aims to improve patients´ knowledge about their disease and support self-management, thus leading to better treatment adherence and health-promoting behaviour [20]. As repetition is a key element of memory formation [21] and spaced retrieval of information improves long-term memory formation [22], some telemedical interventions involve frequent coaching sessions in regular intervals [18]. However, whether repetitive telecoaching sessions are necessary is unclear; and the evidence of optimal duration and frequency of telecoaching session in heart failure is still sparse.

The aim of our study was to observe the short- and long-term effects of a single COVID-19-specific telecoaching module in a cohort of 419 chronic heart failure (CHF) patients already participating in a combined tele-monitoring and telecoaching programme for CHF during the COVID-19 pandemic. We specifically focused on changes in knowledge and reported behaviour over time, but also assessed effects on perceived health, worries and social impact as well as need for medical contacts including COVID-19 infections.

## Methods

### Study design and inclusion criteria

This study is a longitudinal, single-arm observational study, observing the impact of a single COVID-19-specific telecoaching module in heart failure patients over time. This longitudinal analysis complements the analysis of the short-term effects of telecoaching compared to sole written transmission of information [23] (COHIRAB).

All study participants were already participating in a combined tele-monitoring and telecoaching programme for patients with CHF named mecor^®^ [24], prior to study inclusion. In this population of heart failure patients at high risk of COVID-19 complications, it was not deemed ethical to withhold the COVID-19-specific telecoaching session from participants, abolishing the possibility of a randomised controlled trial. Participants for this COVID-19-specific longitudinal study were selected based on their first telecoaching session taking place between April 3rd and 9th 2020 during their pre-scheduled routine telecoaching call.

### Ethics

The study conformed to the ethical standards of the Declaration of Helsinki and was approved by the local ethics committee. Informed consent to participate in the tele-health programme, including data collection and presentation for research purposes was obtained from all subjects. The scientific analysis of the telehealth programme mecor [24], including this substudy, was registered at the German Clinical Trial Register with the identifier DRKS00026197. Personal data was processed in accordance with Directive 95/46/EC (GDPR; supplement a).

### Study population and telehealth programme

All study participants were already participating in a combined tele-monitoring and telecoaching programme for patients with CHF in Germany named mecor^®^ [24]. The programme consisted of regular one-to-one telecoaching sessions every 6–12 weeks conducted by specially trained tele-nurses about CHF-related topics, such as adherence to medication, regular physical activity, balanced nutrition and fluid intake as well as vaccinations against influenza and pneumococci. Furthermore, the programme included daily monitoring of CHF signs and symptoms (e.g., dyspnoea, oedema, weight), which were captured by the patients and transmitted to the telehealth centre for evaluation.

In the telemedical centre, patients´ data was analysed using an automatic validity check and pre-specified rules to identify imminent decompensation (e.g. weight gain, repeated worsening of symptoms). In case of an IT-alert, a telenurse reviewed the alert and contacted the patient by telephone using a standardised interview to verify the validity of the alarm and to assess the patients´ condition. The telenurses advice followed a protocol with escalating behavioural interventions, taking into consideration individual patients´ factors, and recommended general behaviour changes (reduction of salt or fluid intake, exercise or rest), adherence to the prescribed medication, taking standby or on demand (diuretic) medication, or seeking physician advice, in a routine or emergency visit.

This combined telecoaching and telemonitoring programme was offered to patients suffering from heart failure who were hospitalised for acute heart failure in the 18 months prior (ICD codes I50*, I11.0*, I13.0*, I42.0). Exclusion criteria for the programme included severely impaired hearing or sight, dementia, schizophrenia, dependency syndromes, severe chronic kidney disease (Stage 4 and 5) or with very high level of nursing care requirement.

### Covid-19 telecoaching and questionnaire

Between April 3rd and 9th 2020, all study participants (*n* = 419) received a one-to-one COVID-19-specific telecoaching module instead of their routine telecoaching call; early April was the earliest possible time following ethics approval and training of tele-nurses. The COVID-19-specific module consisted of a standardized interview assessing knowledge about COVID-19 infection, preventive behaviour and risk perception (supplement b). Knowledge was assessed by asking open questions e.g. ‘Do you know symptoms associated with a potential Corona-infection?’ and summarised as the number of items recalled. The questionnaire was followed by a coaching module informing patients about symptoms of COVID-19 infections, risk reduction strategies and recommended behaviour in case of infection based on evidence and public health advice available in March 2020 (supplement c). The telecoaching session was followed by a written summary of the conveyed information (supplement d), that was sent to the patients via mail after completion of the coaching session.

To assess short-term effects of the COVID-19-specific telecoaching module, all participants were contacted again by a tele-nurse between April 20th to 30th 2020 (2.5 ± 0.4 weeks after the pre-education interview) and questioned the same standardized interview with additional questions regarding any change in behaviour since the last call (supplement b). Behaviour changes were assessed through asking participants direction questions e.g. Do you receive no/limited visitors? Whilst perceived risk was assessed on a categorical scale.

Between April and November 2020, the regular telemonitoring and telecoaching programme continued, including regular telecoaching calls on an individual basis every 6–12 weeks. These telecoaching calls addressed general issues concerning heart failure but did not routinely cover COVID-19-specific topics. On patient’s request, further COVID-19-specific advice in line with previous telecoaching was offered.

For a long-term evaluation of the COVID-19-specific telecoaching module all participants of the short-term follow-up were assessed again between November 11th 2020 and January 29th 2021 (33.9 ± 2.2 weeks; at the rise of the 2020/2021 winter COVID-19 wave) using the same standardised questionnaire as at short-term follow-up (supplement b). At this time, and due to rapid scientific developments, an additional COVID-19 symptom (loss of taste and smell) was added, as well as a question on patients’ intention to receive influenza vaccination. Information on COVID-19 vaccination was not collected as it was only licensed in Germany at the tail end of this study.

### End points

The primary endpoints of this study were difference in knowledge on COVID-19-related risk reduction measures and difference in self-reported behaviour change between pre-education, short- and long-term follow-up. Secondary endpoints were (1) difference in knowledge of COVID-19-specific symptoms, (2) difference in knowledge regarding reasons for seeking medical attention and contact methods for medical attention, (3) difference in actual number of medical contacts and number of COVID-19 infections as well as (4) change in self-reported psychological impact of COVID-19.

### Statistical analysis

Individual patients’ answers to the standardised questionnaire were collected by the tele-nurse during the interview and collected in the electronic health records database in the telemedical centre. The data were analysed using Prism Graphpad v9 and R according to the study design. Investigators who were not part of the data controller’s organization were provided with anonymized data. All patients were included in the analysis and individual data-points were excluded if no answer was recorded.

For continuous variables, a paired two-sided t-test was used. Fisher’s exact test was applied for categorical data. For non-parametric samples, Mann-Whitney was applied to non-repeated measures whilst Skillings-Mack was used for repeated measures. The Benjamini-Krieger-Yekutieli procedure with a false discovery rate of 0.05 was applied to all statistical tests to account for multiple comparisons. P-values below 0.05 were considered statistically significant.

## Results

**Fig. 1:**
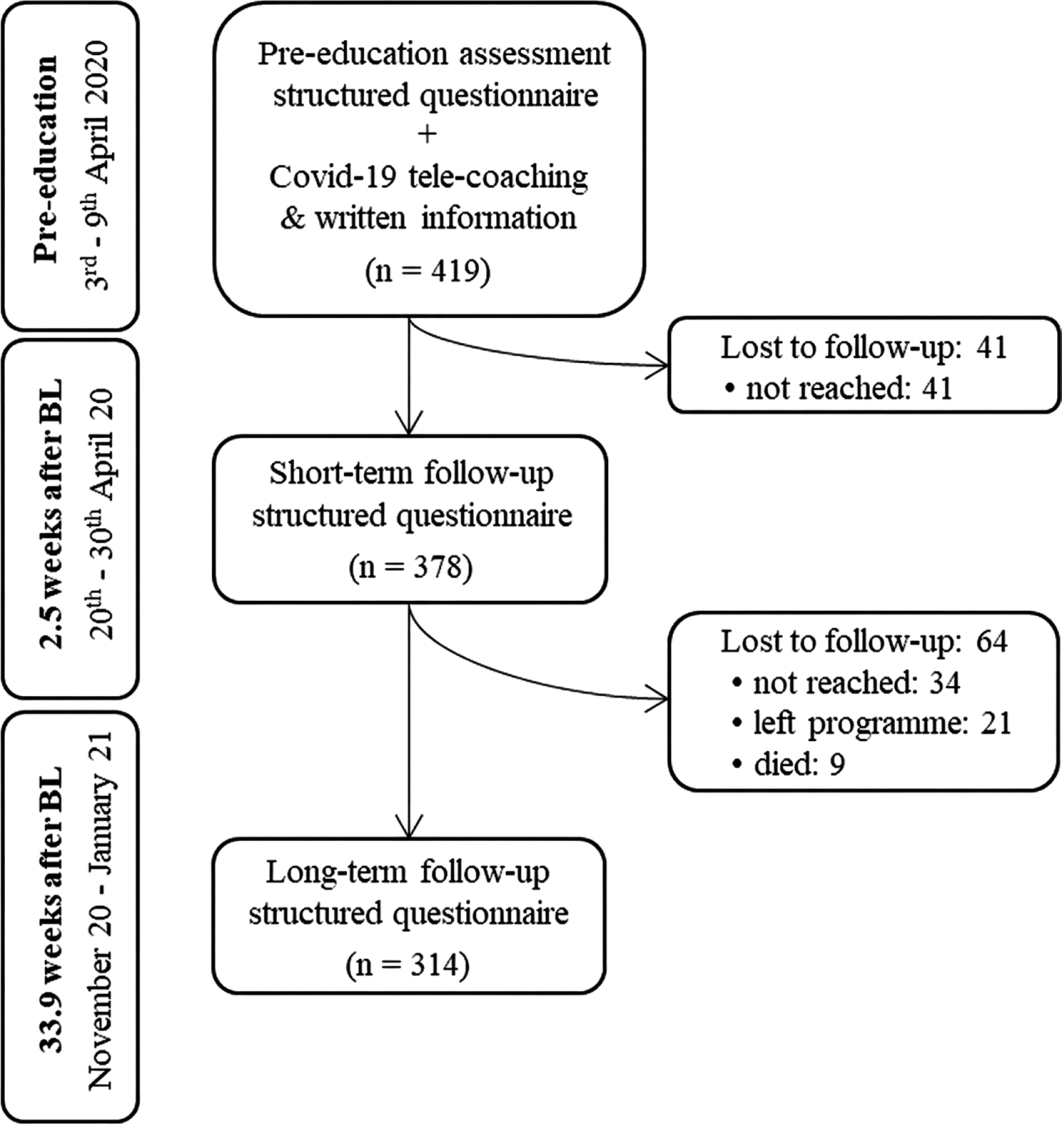
Study flow summary of the observational study design and time-points

Of the initial 419 participants who received the COVID-19-specific telecoaching module during early April 2020, 378 participated in the first follow-up (2.5 ± 0.4 weeks from first telecoaching), whereas 41 participants were not reached. The second follow-up was completed by 314 participants, while 64 were not reached after 33.9 ± 2.2 weeks from the baseline telecoaching (Fig. 1) during November 2020 through January 2021.

### Baseline characteristics

Baseline clinical characteristics of the study population are shown in Table 1. The mean age was 76 ± 9.4 years with 64% of participants being male. Most patients had multiple comorbidities including 88% with hypertension, 44% with diabetes mellitus, and 48% with chronic respiratory disease. All patients suffered from CHF with a New York Heart Association (NYHA) class I-IV, mostly II and III (38% and 57% respectively, Table 1). There was no difference between patients who completed all three time-points and those lost to follow-up for both patient characteristics and questionnaire answers pre-education (data not shown).

**Table 1:** Baseline characteristics for all patients included in the study

Patient characteristics		
		*n* = 419
Age (years)	mean ± SD range	76 ± 9.4 40–98
Gender	Male Female	269 (64%) 150 (36%)
Current dyspnoea (NYHA class)	I II III IV	13 (3.1%) 161 (38%) 239 (57%) 6 (1.4%)
Health perception (EQ-5D-5 L)	mean ± SD	61 ± 23
Respiratory disease ^a^		191 (48%)
Diabetes ^b^		185 (44%)
Hyperlipidaemia		245 (65%)
Cardiac arrhythmia		257 (68%)
Hypertension		357 (88%)
Coronary artery disease		253 (63%)
Chronic kidney disease		134 (33%)
Number of co-morbidities ^c^	0 1 2 3 4 5 6 7	4 (1.0%) 10 (2.4%) 51 (12%) 93 (22%) 135 (32%) 78 (19%) 35 (8.4%) 13 (3.1%)
Highest academic qualification	University Degree Abitur (University entrance qualification) Realschule (intermediate school diploma) Hauptschule (school leaving diploma) No school certificate	31 (7.4%) 35 (8.4%) 97 (23%) 245 (58%) 11 (2.6%)

^a^ Respiratory disease includes: asthma and/or chronic obstructive pulmonary disease (COPD), silicosis, fibrosis; ^b^ diabetes is defined as history of type 1 or 2 diabetes mellitus independent of management; ^c^ sum of co-morbidities (respiratory illness, diabetes, hyperlipidaemia, cardiac arrhythmia, hypertension, coronary artery disease, chronic kidney disease) for each patient. Where it was not recorded if a patient has a specific co-morbidity the data point was excluded. Due to rounding, total may not add up to 100%

### COVID-19-specific risk reduction measures and behavioural change

**Fig. 2:**
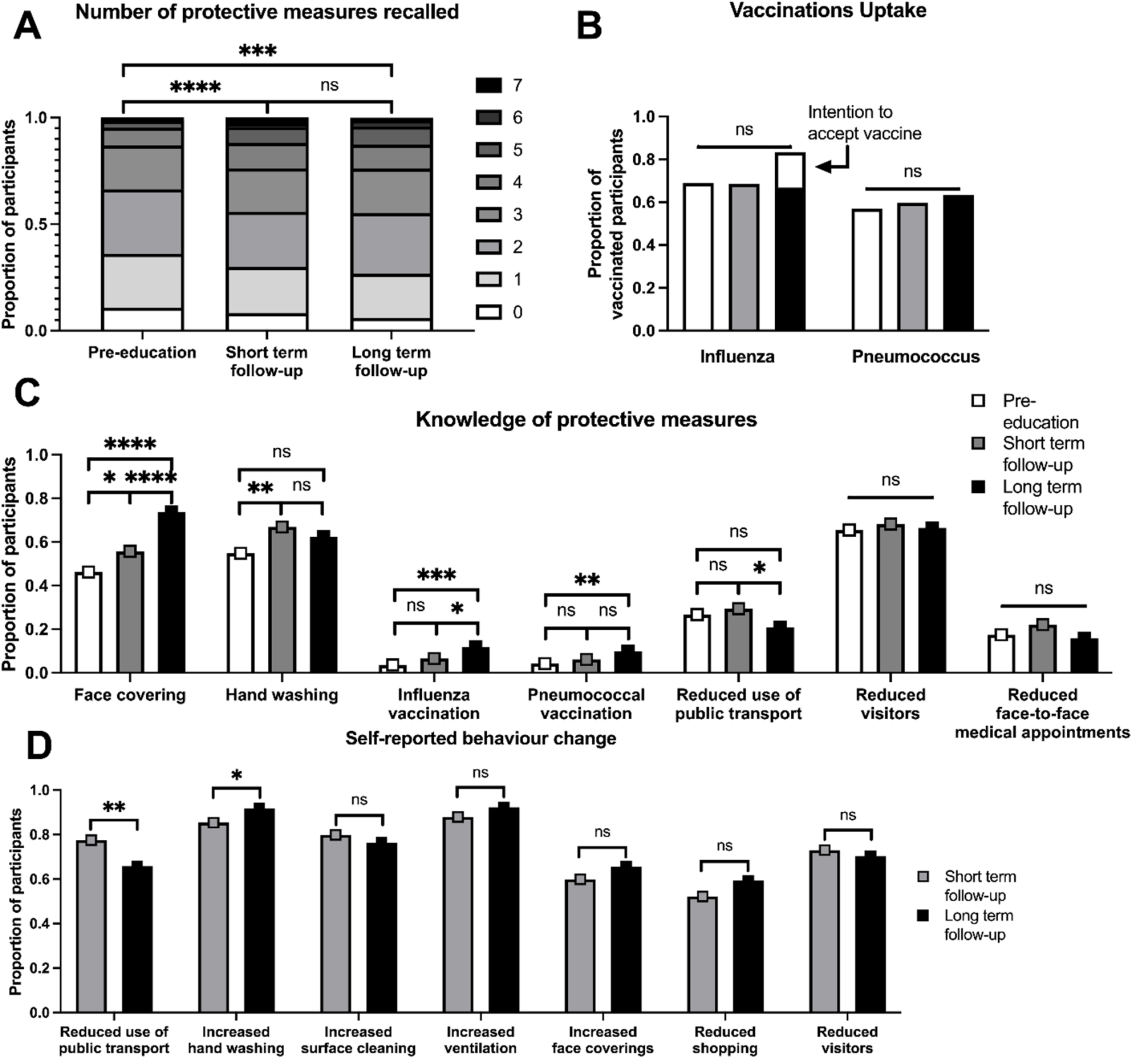
Knowledge of COVID-19-specific risk reduction measures and behavioural change. **A**: Number of COVID-19-specific risk reduction measures recalled pre-education, at short- and long-term follow-up. **B**: Proportion of vaccinated patients pre-education, at short- and long-term follow-up. **C**: Individual COVID-19-specific risk reduction measures recalled pre-education, at short- and long-term follow-up. **D**: Individual behavioural changes reported at short- and long-term follow-up. Ns = not significant, *: p < 0.05, **: p < 0.01, ***: p < 0.001, ****: p < 0.0001

Knowledge about COVID-19-specific risk reduction measures were assessed through open questions and only unprompted patients´ answers were evaluated. As first primary endpoint, we analysed overall knowledge about COVID-19-specific risk reduction measures. Prior to COVID-19-specific telecoaching, 34% of patients named at least three protective measures, whilst at short- and long-term follow-up, these numbers rose to 44% and 45% of patients, respectively. On the contrary, 36% of patients spontaneously recalled zero or one preventive behaviour prior to telecoaching, 30% at short-term and 27% at long-term follow-up. The difference of recalled items between the three time points (pre-education, short-term and long-term follow-up) was significant (*p* = 0.0002, Fig. 2a).

The individual single preventive behaviours recalled by the patients differed slightly between the three time points (Fig. 2c). While knowledge about the importance of reducing medical contacts and household visitors did not significantly change over time, knowledge about regular hand washing increased from 55% to 67% and 62% (*p* = 0.003) and about the importance of using face masks from 46% to 56% to 74% (*p* < 0.001). On the contrary, avoidance of public transport was mentioned by 27% of patients before coaching and 29% or 21% at short- and long-term follow-up, respectively (*p* = 0.041).

Knowledge about the preventive importance of vaccinations, both pneumococcal and influenza, was low prior to education (4% for each), but increased significantly over time to 6% and 10% (*p* = 0.012) for pneumococcal and to 7% and 12% (*p* = 0.001) for influenza vaccine at short- and long-term follow-up, respectively. In this high-risk group of chronic heart failure patients, 57% were vaccinated for pneumococci and 69% for influenza before telecoaching. Whilst influenza vaccination rates remained stable, numerically more patients reported being vaccinated for pneumococci over time. At the time of long-term follow-up, 50% of those patients not vaccinated for influenza, stated that they would accept a vaccine if offered (Fig. 2b).

As second primary endpoint we assessed differences in self-reported behaviour change. When questioned directly on actually implemented changes in behaviour, patients reported avoidance of public transport more often at short-term than at long-term follow-up (78% vs. 66%, *p* = 0.0009), whereas self-reported regular hand washing increased from 86% to 92% (*p* = 0.012). Rates of self-reported behaviour change regarding use of face coverings, regular surface cleaning and ventilation as well as reducing household visitors and shopping did not significantly differ over time (Fig. 2d).

### Knowledge of COVID-19 symptoms and medical contact

**Fig. 3:**
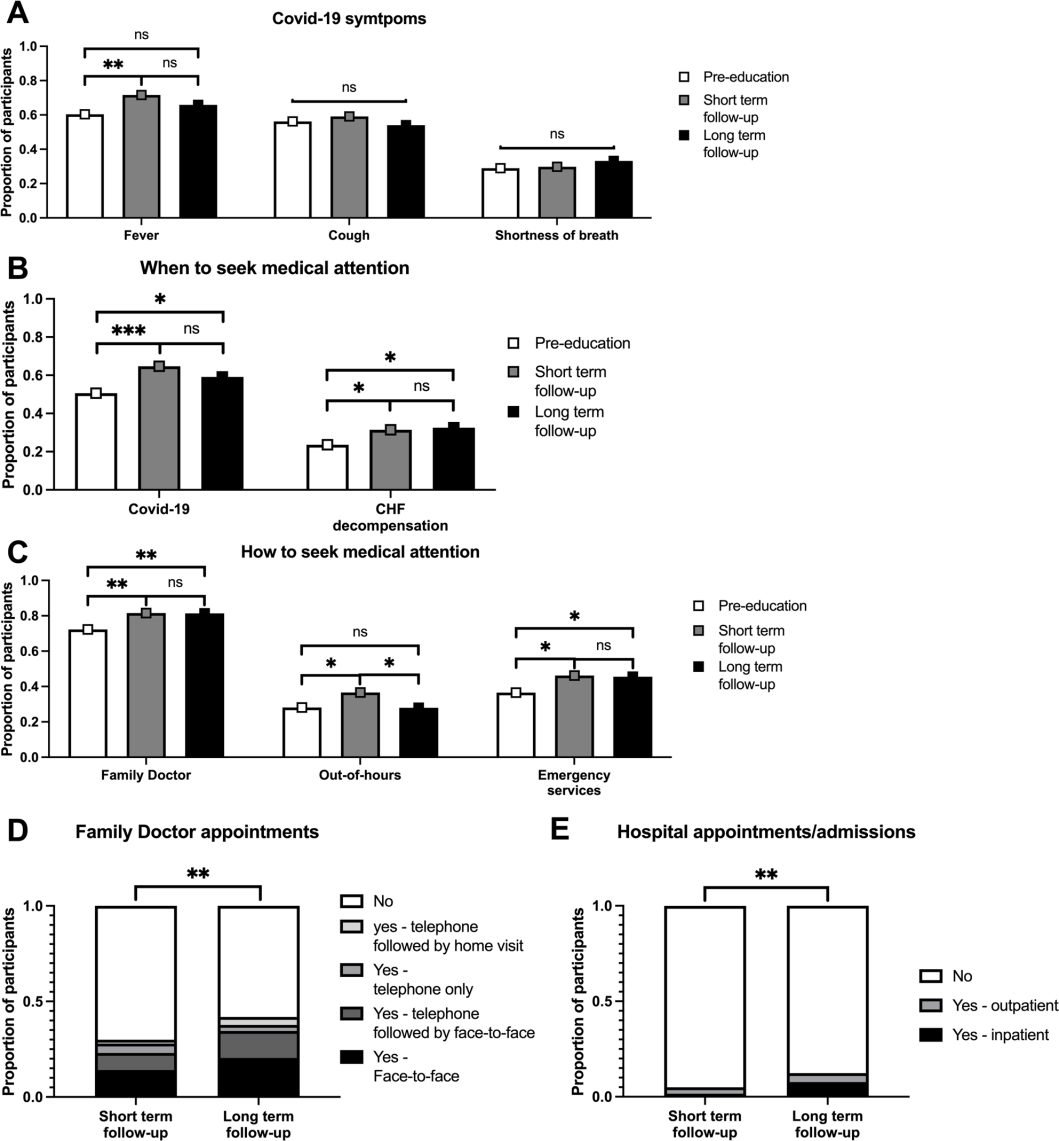
Knowledge of COVID-19 symptoms and medical contact **A**: Individual COVID-19 symptoms recalled pre-education, at short- and long-term follow-up. **B-C**: Knowledge about when (**B**) and how (**C**) to seek medical attention pre-education, at short- and long-term follow-up. **D-E**: Self-reported medical contacts in the previous three weeks at the family doctor (**D**) and at the hospital (**E**) at short- and long-term follow-up. Ns = not significant, *: p < 0.05, **: p < 0.01, ***: p < 0.001

Besides assessing knowledge on preventive behaviours, we assessed patients´ knowledge about the COVID-19 disease as well as the right approach to treatment. The number of spontaneously recalled COVID-19 symptoms did not differ between the three different time points (pre-coaching, short- and long-term follow-up) analysed. Fever was more frequently recalled as typical COVID-19 symptom after telecoaching, especially at short-term follow-up (60% to 72% to 66%, *p* = 0.004, Fig. 3a), while shortness of breath and cough were mentioned as COVID-19-specific symptoms similarly over time.

During the follow-up period, patients were significantly more aware of when to seek medical contact. This was the case both for COVID-19 infection (mentioned spontaneously by 51% of patients before coaching, 65% at short-, and 60% at long-term follow-up, *p* = 0.0004) as well as for decompensation of their underlying heart failure (mentioned by 24%, 32% and 33% of patients at the three time points, respectively, *p* = 0.017, Fig. 3b). Additionally, spontaneous recall of how to reach their primary care doctor increased significantly from 72% to 82% to 81% (*p* = 0.002), whilst knowledge of out-off-hours services increased from 28% to 32% at short term follow-up before falling back to 28% (*p* = 0.016, Fig. 3c).

In addition to knowledge and behaviour changes, we assessed need for health care interaction during the COVID-19 pandemic in this cohort of heart failure patients. The number of self-reported medical contacts during the previous three weeks increased between the short- and long-term follow-up. This increase affected both community doctors as well as hospital visits (Fig. 3d and e). 30% of patients at short-term follow-up and 42% at long-term follow-up reported visiting the primary care physician (*p* = 0.006). Hospital attendance was reported by 4.9% of patients at short-term and 12% at long-term follow-up (*p* = 0.001).

The overall number of SARS-CoV-2 infections in the study population during the study period was low (1.2%). A total of five patients tested positive – one patient reported a positive result in April 2020 whilst the remaining four patients reported a positive test during long-term follow-up. There was no statistical difference in the proportion of PCR-confirmed SARS-CoV-2 infections over time.

### Perception of health risk for a severe COVID-19 infection and subjective health status

**Fig. 4:**
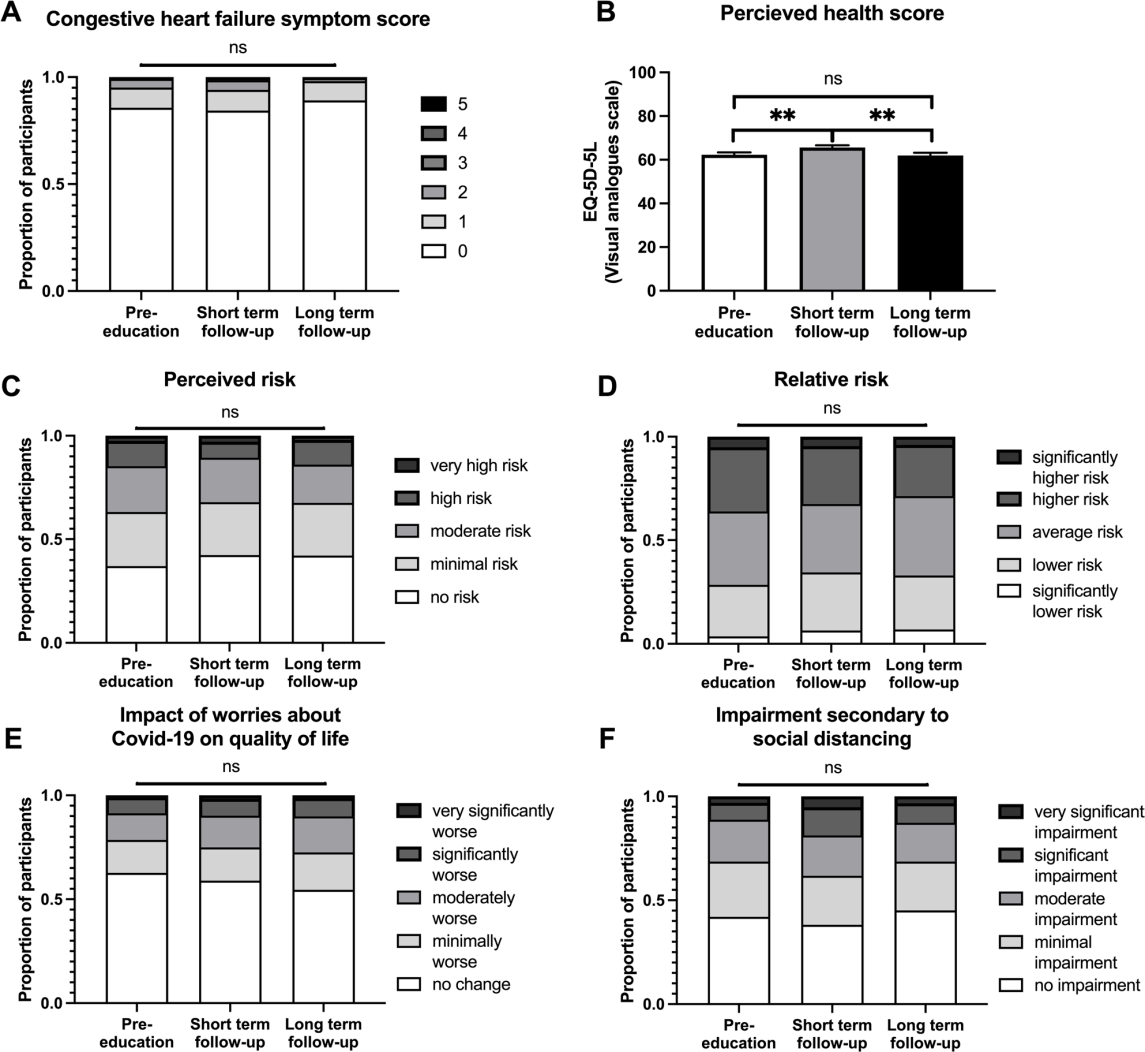
Subjective risk awareness and perceived health status **A**: Self-perceived congestive heart failure symptom score (consisting of existing oedema, need for an additional pillow at night, cough, shortness of breath and tiredness/ fatigue) pre-education, at short- and long-term follow-up. **B**: Self-perceived health score (as per EQ-5D-5 L-scale) pre-education, at short- and long-term follow-up. **C-D**: Self-perceived absolute (**C**) and relative (**D**) risk of COVID-19 pre-education, at short- and long-term follow-up. **E**: Impact of worries about COVID-19 on quality-of-life pre-education, at short- and long-term follow-up. **F**: Impairment due to social distancing pre-education, at short- and long-term follow-up. Ns = not significant, **: p < 0.01

As secondary endpoint of the study, patients were asked to assess their self-perceived health status as per EQ-5D-5 L-scale and using a heart failure symptom score consisting of peripheral oedema, need for an additional pillow at night, cough, shortness of breath and fatigue. Whilst the heart failure symptom score did not change over time, patients rated their health as better during short-term follow-up than at any other time point (*p* = 0.045, average score 62 to 66 to 62 on the EQ-5D-5 L-scale, Fig. 4a-b).

Furthermore, patients were asked about their subjective health risk for a severe COVID-19 infection, both absolutely as well as compared to the general, healthy population. Pre-education, only 2.5% of patients perceived themselves as very high-risk for a severe COVID-19 infection, while 37% considered themselves at no risk. These perceptions did not significantly change at short- or long-time follow-up, with 2.9% and 1.9% of patients regarding themselves at very high-risk and 42% and 42% as no risk at short- and long-term follow-up, respectively. Similarly, perceived risk compared to the general population was considered very high by 5.0% pre-education and 4.5% at short-term and 4.0% at long-term follow-up (Fig. 4c-d).

Finally, the impact of worries about COVID-19 on the quality of life as well as the impairment secondary to social distancing was evaluated over time. There was no difference in impairments due to social isolation or concern about COVID-19 over time (Fig. 4e-f).

## Discussion

In this study, we specifically focussed on assessment of the telecoaching aspect of an established combined telecoaching and telemonitoring programme. Furthermore, we assessed knowledge and self-perceived risk assessment of heart failure patients in regard to the COVID-19 pandemic.

In this longitudinal single-arm observational study, we observed a gain in knowledge about COVID-19-specific preventive behaviours at short-term after a COVID-19-specific telecoaching session compared to before the telecoaching session. Nevertheless, our data also shows that despite regular coverage of COVID-19-related topics in the media, overall knowledge of typical COVID-19 symptoms and protective measures was moderate to poor in our cohort of chronic heart failure patients. This is concerning, given the high risk status of this heart failure population [5] and supports the claim for additional targeted education in high risk patients.

We found that even though most COVID-19-specificknowledge persisted over time, some particular knowledge seemed to fade. For instance, knowledge items of reduced use of public transport and how to contact out-off-hours medical support increased after telecoaching on short-term follow-up but declined again on long-term follow-up. In general, even half a year after the telecoaching module and with concurrent public-health messaging, participants’ overall knowledge of protective measures was high.

On the contrary, two particular knowledge items, face covering and vaccination uptake, were more often mentioned in January 2021 compared to April 2020. This finding might at least partially be influenced by concurrent public health campaigns and discussion in the media during the time of our study. During the pandemic, daily reports about the COVID-19 disease and protective measures contributed to patients´ knowledge and behaviour changes. Particularly, this applies to the knowledge of the importance vaccinations, a topic broadly discussed with emerging COVID-19 vaccines. This also applies to the use of face coverings, fundamental in public precaution recommendations. It is not possible in our observational study to separate the public health campaigns from the COVID-19 specific telecoaching.

Besides changes in knowledge about COVID-19 preventive behaviours, we also assessed implementation of recommended behaviours. Similar to the relatively little differences of knowledge seen between short- and long-term follow-up, we saw only few significant differences in self-reported behaviour changes (hand washing and use of public transport). These findings are reassuring, as they suggest a persistent knowledge and behaviour change. However, they are also concerning, as despite the telecoaching and public health campaigns, some patients did not change their behaviours. Only increased hand washing was more frequently mentioned at long-term follow-up, suggesting very little behavioural change without active encouragement. Potentially, telemedical interventions focusing specifically on encouraging behaviour change besides improving knowledge might be more efficient in this regard.

Furthermore, we assessed frequency of outpatient and in-hospital medical contacts. Interestingly, the number of reported medical contacts in the three weeks prior to each questionnaire increased between the short- and long-term follow-up in our study. This might be the result of deferred routine appointments during the early phase of the pandemic and subsequently increased numbers of re-scheduled routine visits but also of more urgent presentations and hospitalisations due to excessive postponement [9]. Alternatively, increased numbers might be a reflection of seasonal variation of heart failure hospitalisations, which have been known to increase during winter [23, 24].

A further finding is that our cohort of heart failure patients did not perceive themselves as high risk for a severe COVID-19 infection, both absolutely as well as compared to the general, healthy population. This (skewed) perception was not prevented by the broad public health campaign at the time and, unfortunately, did not change after the COVID-19-specific telecoaching session. Similarly, an explorative study in US residents in March and April 2020 showed that adults with pre-existing conditions underestimate their risk of dying from COVID-19 [25]. This applies both for the absolute and relative fatality risk perception [25]. Our finding is also consistent with the literature on self-perceived health-risk in heart failure: Patients with heart failure underestimate the health risks associated to their chronic condition, especially if they are older [26–28]. These results are concerning, because health promoting and disease preventing behaviours are often driven by risk perception [25, 29].

Finally, another important aspect related to self-perceived risk was the assessment of worries related to the COVID-19 pandemic and the effect of the COVID-19-specific telecoaching on these worries. Interestingly, worries related to COVID-19 did not increase after compared to before telecoaching or over the course of the pandemic. Similarly, patients reported consistent impairment due to social distancing, despite the severe government restrictions during lockdown. This is in line with reports from the general population in Germany [30].

## Limitations

The main limitation of our study is the missing control group, limiting the ability to attribute observed changes solely to the telecoaching module. Given the lack of a control group, our results have to be interpreted with caution, as additional information sources besides the telecoaching sessions, e.g. the media, might have influenced patients´ knowledge on COVID-19 and their risk behaviour. Still, it was not deemed ethically acceptable to withhold the COVID-19-specific telecoaching from high-risk heart failure patients during the early phases of the COVID-19 pandemic. Future studies evaluating the effects of telecoaching should address these limitations either by stepped-wedge or cluster-randomized approaches.

Furthermore, our study was limited to patient interviews without access to secondary data sources. This study design carries the risk of recall and social desirability bias. A proportion of patients will have additional challenges with memory in view of their advanced age and co-morbidities such as depression. During the interviews, patients could consult doctor’s letters or notes and vaccination cards, but the study team did not have access to these. This limitation especially applies to details about medical visits and vaccination status as well as the numbers of COVID-19 infections. To address these limitations, future research using objective behavioural and clinical outcome measures, such as health insurance claims or wearable activity data is needed.

Finally, as our study focussed on telecoaching in heart failure patients, generalizability is limited and further studies are needed to analyse telecoaching in regard to other chronic conditions. There is a need for further (randomised controlled) studies evaluating the ideal intensity of telemedical interventions.

## Conclusion

Our longitudinal observational study of chronic heart failure patients attending a COVID-19-specific telecoaching during the first wave of the COVID-19 pandemic indicates a benefitof the telecoaching on knowledge about the disease and complying with risk reduction measures, which seems to persist over time. However, even after specific telecoaching and concurrent public health campaigns, knowledge about the disease and risk reduction measures in our high-risk population of chronic heart failure patients remained poor, highlighting the need for further studies on the topic. Our findings underline that repetitive telecoaching may be necessary to substantiate disease knowledge and to solidify persistent behaviour changes.

## Supplementary Information

Below is the link to the electronic supplementary material.

Supplementary Material 1

## Data Availability

The datasets analysed during the current study are not publicly available due privacy constraints.

## Abbreviations

CHF: Chronic heart failure
COHIRAB: COrona Hlgh Risk Awareness and Behaviour Study
COVID-19: Corona Virus Disease 2019
EQ-5D-5 L: EuroQol five-dimensional five-level
GDPR: General Data Protection Regulation
NYHA class: New York Heart Association class
SARS-CoV-2: Severe Acute Respiratory Syndrome coronavirus 2
