# Supplementary material for: Observation of risk perception, knowledge and behaviour related to covid-19 in heart failure patients enrolled on a telecoaching program
Source: BMC Med Inform Decis Mak. 2026 Jun 22;26:227. doi: 10.1186/s12911-026-03613-y (PMC13292520; doi:10.1186/s12911-026-03613-y)
Supplement: Supplementary file 1 — Supplementary Material 1 [file 12911_2026_3613_MOESM1_ESM.pdf]

Supplement

Supplement a: Data protection and information security

For the COHIRAB study, personal data from patients already participating in the mecor® tele-health programme (participant data) were analysed with respect to their COVID-19 risk perception and behaviour. For these data, Health Care Systems GmbH (HCSG) is the data controller within the meaning of Article 4 (7) of Regulation (EU) 2016/679 of the European Parliament and of the Council of 27 April 2016 (General Data Protection Regulation, GDPR). HCSG remains solely responsible for the processing of participant data in accordance with Article 24 GDPR.

Before participating in mecor®, patients gave their explicit consent to the processing of their personal data according to Articles 6 (1) (a) and 9 (2) (a) GDPR. At the time of enrolment into mecor®, patients were informed according to Article 13 GDPR. The information comprised, for example, details about the data controller, the participants' rights under GDPR, the purpose of data processing together with categories, types and sources of data. In particular, patients were informed that their data may also be processed for the purpose of research.

Patients' electronic health records are kept on the mecor® software, which is certified as a medical device according to MDD 93/42. The information security management system of HCSG is certified according to ISO/IEC 27001.

Personal data were extracted and analysed by the data controller' staff in the data controller's highly secure data centre. Investigators that were not part of the data controller's organization were provided with anonymized data for further statistical analyses.

Supplement b: Questionnaire for telephonic interview

This standardized questionnaire assessed knowledge about preventive and risk reduction measures by open, unprompted questions. If the patient was not able to name a risk reduction measure spontaneously, it was considered as not known and no answer was prompted. Behaviour change, current health status, and medical attendance were evaluated with prompted questions offering a battery of choices for each item. All items were self-reported. The original questionnaire was provided in German.

Questionnaire:

Have you changed your behaviour since you received information?[+]

- Do you receive no/limited visitors?	YES/NO/UNKNOWN
- Do you avoid public transport?	YES/NO/UNKNOWN
- Do you shop still shop yourself?	YES/NO/UNKNOWN
- Do you thoroughly wash your hands?	YES/NO/UNKNOWN
- Do visitors wear a mask?	YES/NO/UNKNOWN
- Do you regularly ventilate?	YES/NO/UNKNOWN
- Do you regularly clean surfaces?	YES/NO/UNKNOWN

Have you been in contact with medical professionals in the last 3 weeks?[+]

- Community doctor?	NO/YES – TELEPHONE/ YES – SURGERY AFTER TELEPHONE/ YES – HOME VISIT/ YES – SURGERY DIRECT
- Out-of-hours doctor?	NO/ YES – TELEPHONE/ YES – SURGERY AFTER TELEPHONE YES – HOME VISIT/ YES – SURGERY DIRECT
- Hospital:	NO/ YES – OUTPATIENT/ YES – INPATIENT

What was the reason of the interaction? [Δ]

ROUTINE / CORONA / INFLUENZA / COLD / HEART / OTHER

Details of interaction? [Δ] FREE TEXT

Do you have regular check-ups with your cardiologist? YES/NO/UNKNOWN

How do you perceive your risk from the Corona-virus?

MINIMAL RISK / LOW RISK / MODERATE RISK / HIGH RISK / VERY HIGH RISK

How do you perceive your risk relative to the average person?

SIGNIFICANTLY LOWER RISK / LOWER RISK / AVERAGE RISK / HIGHER RISK / SIGNIFICANTLY HIGHER RISK

Have concerns or fears about Corona impacted on your quality of life?

NO IMPACT / MINIMALLY WORSE / MODERATELY WORSE / SIGNIFICANTLY WORSE / VERY SIGNIFICANTLY WORSE

How impaired do you feel through the social isolation secondary to Corona?

NO IMPAIRMENT / MINIMAL / MODERATE / SIGNIFICANT / VERY SIGNIFICANT IMPAIRMENT

How do you perceive your health today on a scale for 1 (worst) to 100 (best)?

Have you been tested for Corona-virus?

NO / YES & POSITIVE / YES & NEGATIVE / YES & UNKNOWN

Have you had contact in the last two weeks with someone who has Coronavirus? YES / NO

Do you know how you can protect yourself? (Patient lists measures unprompted).

- Pneumococcal vaccine YES / NO
- Influenza vaccine YES / NO
- Reduced visitors YES / NO
- Reduced use of public transport YES / NO
- Reduced medical interactions YES / NO
- Hand washing YES / NO
- Face masks YES / NO

Do you know symptoms associated with a potential Corona-infection? (Patient lists symptoms unprompted).

- Fever YES / NO
- Shortness-of-breath YES / NO
- Cough YES / NO
- loss of smell or taste* YES / NO

Do you know when to seek urgent medical attention? (Patient lists reasons unprompted).

- Corona-infection: fever > 38°C, worsening shortness of breath or cough YES / NO
- CCF decompensation: signs YES / NO

Do you know how to seek medical attention? (patient lists contacts unprompted for each of the below)

- Community doctor YES / NO
- Out-of-hours via 116 117 YES / NO
- Emergency via 112 YES / NO

Are you currently vaccinated against Influenza? YES / NO / UNKNOWN

Are you currently vaccinated against Pneumococcus? YES / NO / UNKNOWN

If not vaccinated: are you planning to get vaccinated against Influenza?* YES / NO / UNKNOWN

+ *Question added at short- and long-term evaluation*

Δ *Question only available at short-term evaluation*

* *Question added at long-term evaluation*

Supplement c: Tele-coaching module

Risk awareness:

Advising patients that they are in a high-risk group. Their outcome of an infection by SARS-CoV-2 could be particularly poor. Reference to individual risk factors e.g. age, hypertension, diabetes, coronary artery disease, respiratory illness, chronic kidney failure, immunosuppression (e.g. steroids).

Risk reduction – physical distancing

- Do not receive guests
- If visitors are necessary, ask them to wear a face mask
- Avoid hand shaking or similar physical contacts
- Don’t leave the house, particularly avoid public transport
- Ask for shopping to be dropped off at your door
- Stay in contact with family/friends via telephone
- Don’t visit medical facilities (unless in case of emergency); ring ahead to ask if a pre-planned appointment is to be kept

Risk reduction – hygiene

- Wash hands regularly with soap
- Use disposable tissues once and discard immediately
- Ventilate rooms regularly
- Clean surfaces
- Cough/sneeze into your elbow

COVID-19 symptoms/signs and when to seek medical attention

- Fever > 38°C and worsening shortness of breath and/or worsening cough
- Discuss signs of cardiac decompensation

Seeking medical attention

- If concerned: first make telephone contact with your community doctor, do not walk directly to the surgery

- If urgent and unable to ring community doctor, call out-of-hours service at 116117
- In emergencies ring 112

Vaccinations

- Use next unavoidable visit to the community doctor to update pneumococcal and influenza vaccinations
- However, physical distancing is the priority

Supplement d: Written information

The new corona-virus – this is how you can protect yourself.

You are at high risk!

The risk of severe illness rises above the age of approx. 50 years with increasing age. Especially older people can have a severe illness following infection. People with hypertension, diabetes, heart-disease, lung-disease (esp. smokers), liver disease, kidney disease, cancer or with a weakened immune system have a higher risk of severe illness. The risk is especially high if someone has multiple of these conditions.

Corona-virus is commonly transmitted through:

- Close and prolonged contact
- Aerosols (sneezing or coughing)
- Via touching contaminated surfaces

How to protect yourself:

Stay at home!

- Cancel all appointments (no social or sporting clubs, no parties, no hair-dressers appointments or similar).
- Avoid routine medical visits. Call the surgery to find out if the visit in required under the current circumstances
- If concerned over your health contact your doctor over the phone. It may be possible to avoid a physical visit.
- Only leave the house for truly necessary trips, avoid use of public transport.
- If you have the leave the house, avoid touching surfaces and pay particular attention not to touch your own face. Wash your hands as soon as you get home.
- Restrict personal visits to a minimum. Stay in contact over the phone or internet.
- Let family, friends, or acquaintances bring supplies; let them drop off shopping bags in front of your door. Wash your hands after touching the door and food.

- If a visit to your house is required, ask the visitor to wear a face covering (mask) and to avoid touching things in your home, to keep you safe. Keep your distance from visitors and ventilate the room well.

Keep your distance!

- Avoid close contact greetings (hand shaking, kisses)
- Keep a distance of 2 m to other people

Clean door handles and other surfaces:

- Clean door handles, light switches, mobile phones, key boards and other surfaces e.g. with window/glass cleaner

Wash your hands regularly and thoroughly!

- Especially, when coming home, after having visitors, before eating, after using the bathroom, after waste-handling
- Use soap; if possible, use liquid soap
- Correct handwashing takes a minimum of 30 seconds: rub your hands until foam develops, spread this foam to include the space between your fingers, underneath your nails, and go down to your wrists
- Dry your hands thoroughly with a clean towel; if possible, use a disposable hand towel or kitchen paper

How to recognise an infection by the novel corona-virus

Typical symptoms:

- Fever (above 38°C)
- Worsening shortness-of breath
- Worsening dry cough

Also, possible symptoms:

- Loss of the sense of smell or taste
- Fatigue (worse than usual)
- Non-specifically feeling unwell e.g. joint pains

- Diarrhoea

Seek immediate medical attention in the following circumstances:

- Fever and worsening shortness of breath OR
- Fever and worsening cough OR
- Worsening shortness of breath and feeling generally unwell (even without fever)

In these circumstances, do not directly visit your doctor or the hospital, but call your community doctor or 116 117. Call 112 in case of acute shortness of breath.
